# Using an Unbiased Coexpression Network to Reveal Cross‐Talking Pathways of Phosphoinositide‐3‐Kinase Regulatory Subunit 1 in Skin Aging and Rejuvenation

**DOI:** 10.1096/fj.202402347RRRR

**Published:** 2026-01-16

**Authors:** Zhike Zhou, Sha Sha, Xiangnan Zhou, Chundi He, Ting Xiao, Yan Wu, Fenqin Chen, Le Qu, Hong‐Duo Chen

**Affiliations:** ^1^ Department of Geriatrics The First Hospital of China Medical University Shenyang China; ^2^ Department of Dermatology Shengjing Hospital of China Medical University Shenyang China; ^3^ Department of Dermatology The First Hospital of China Medical University Shenyang China; ^4^ Key Laboratory of Immunodermatology Ministry of Education and Ministry of National Health Commission Shenyang China

**Keywords:** co‐expression network, differentially expressed genes, phosphoinositide‐3‐kinase regulatory subunit 1, skin aging, skin rejuvenation

## Abstract

Skin aging is a highly complex process embracing chronological aging and photoaging. Continuous advancement in the study of skin aging facilitates the innovations of skin rejuvenation therapies. However, the mechanism underlying skin aging remains largely unexplored. Herein, differential expression gene (DEG) analysis identified a gradual down‐regulation of PIK3R1 in aged untreated skin, aged treated skin (treated with intense pulsed light), and young skin. Among 11 964 background genes, thousands of DEGs were identified in all aged/young and PIK3R1‐low/high groups. Coexpression modules were created using weight gene correlation network analysis, showing an enrichment in PI3K/AKT, Rap1, regulating pluripotency of stem cells (rPSC), etc. Furthermore, DEGs with strong relation to skin vitality and PIK3R1 were extracted for network analysis, wherein cross‐talking pathways of PIK3R1 including PI3K/AKT, Rap1, and rPSC were identified. The same cross‐talking pathways were also replicated in aged untreated and treated skin, as implemented by enrichment analyses of DEGs in aged untreated versus young or aged treated skin. According to the area under the curve of 100% and 88%, PIK3R1 possibly predicted skin aging and skin rejuvenation, respectively. RT‐PCR and western blot confirmed the decline of PIK3R1 expression in aged treated and young skin, compared with aged untreated skin. PIK3R1 knockdown led to increased p‐AKT (Ser473) and Bcl‐2, and decreased p‐FOXO1 (Ser256) and MMP‐1, which may be the cause of more resistance to ultraviolet A induced cell senescence, proliferation inhibition, apoptosis, and collagen synthesis decline in PIK3R1 knockdown HDFs. Our study preliminarily elucidates the comprehensive role of PIK3R1 in skin aging, providing a potential new target for skin rejuvenation.

## Graphical abstract

Mechanistic pathways of PIK3R1 in skin aging and rejuvenation were analyzed by bioinformatics approach and Gene Expression Omnibus database. Compared with young skin, PIK3R1 was differentially expressed in aged and IPL rejuvenated skin. Coexpression and network analyses revealed that PIK3R1 jointly participated in skin aging and rejuvenation via PI3K/AKT, Rap1, and rPSC. PIK3R1 knockdown HDFs were more resistant to UVA induced senescence, proliferation inhibition, apoptosis, and collagen decline, which might be caused by alteration of p AKT, p‐FOXO1, Bcl‐2, and MMP‐1.

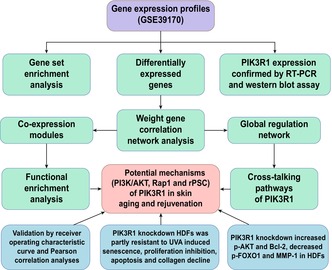

## 1 Introduction

As the largest organ of the human body, skin protects the internal organism from external environmental factors. Like all other organs, skin ages over time [1]. Skin aging has become a worldwide concern because not only does it significantly affect people's appearance, but also it leads to multiple diseases that affect our quality of life. Skin aging includes both intrinsic and extrinsic aging [2, 3, 4]. Different aspects are proposed to explain the mechanism for skin aging, including cellular senescence, ultraviolet (UV) radiation, oxidative stress, telomere shortening, genetic mutations, DNA repair capacity, hormone levels, and so on [2, 3, 4]. As skin ages, it naturally results in thin, dry skin, wrinkles, pigmentation, loss of elasticity, and laxity. In aged skin, a remarkable change is that the proliferation ability of keratinocytes, fibroblasts, and melanocytes is decreased. Meanwhile, the amount reduction and structural changes in extracellular matrix (ECM) proteins such as collagen, elastin, fibrillin, and glycosaminoglycan are also involved in skin aging [4, 5]. For the underlying mechanisms of these alterations, previous studies reported that transforming growth factor‐β (TGF‐β) [6], phosphoinositide 3‐kinase (PI3K) [7], phosphoinositide‐dependent protein kinase‐1 (PDK1) [8] and matrix metalloproteinases (MMPs) [6] played important roles in the process of skin aging. Substantial progress has been made in elucidating the cellular and molecular mechanisms that bring about skin aging. However, the precise mechanism has not been fully clarified.

Skin plays a crucial role in the physical attractiveness of human beings. Intense pulsed light (IPL) technology has been widely used for skin rejuvenation since over 20 years, adhering to the principles of selective photothermolysis [9, 10]. Based on this theory, IPL has shown to be effective in treating the pigmented concerns and the vascular components of skin aging, and over time, have an effect on the collagen itself which improves wrinkles and skin texture [9, 10, 11]. The mechanism may be through the increasing activity of fibroblasts and rearrangement of both collagen and elastin in the ECM. Nevertheless, the underlying mechanism of IPL treatment for aged skin remains unclear. Although fractional laser (nonablative and ablative) [12, 13, 14] and radiofrequency (RF) [15, 16] are also applied for skin rejuvenation. However, the principles are both different from IPL. For example, ablative fractional CO_2_ (AFCO_2_) laser is one of the most used fractional lasers for skin rejuvenation. It creates regions of columnar ablative thermal damage from the epidermis to reticular dermis, known as the microthermal treatment zones (MTZs), which stimulate re‐epithelization via induction of wound healing, collagen production and dermal remodeling [12, 13, 14]. RF is a kind of alternative to skin rejuvenation. In contrast to lasers that target specific chromophore, RF is chromophore independent and has better penetration to the dermis and hypodermis. For example, fractional microneedle RF achieves it clinical effects through penetration to the dermis or even hypodermis and delivery of RF energy into the dermis triggering collagen biosynthesis and remodeling [15, 16]. For the production and remodeling of collagens and other ECM in the process of IPL, RF and fractional CO_2_ laser rejuvenation, the detailed molecular mechanisms are rarely reported and far from fully understood. Therefore, it is not easy to distinguish different treatments from molecular mechanisms. Up to present, it is known that the expression of TGF‐β, MMPs, heat shock protein (HSP) and vascular endothelial growth factor (VEGF) Xu et al. are altered after different treatments and might play important roles in IPL, RF and fraction CO_2_ laser rejuvenation [17, 18, 19, 20, 21, 22, 23]. A recent study revealed that skin aging was associated with a significantly altered expression of 2265 protein coding and noncoding RNAs, of which 1293 became “rejuvenated” after IPL treatment [11]. The microarray RNA sequencing data was accessible through Gene Expression Omnibus under the series accession number GSE39170. In summary, firstly, IPL is one of the most widely used treatments for skin rejuvenation. Secondly, the theory principle of IPL is different from other skin rejuvenation therapeutic approaches such as fractional CO_2_ laser and RF. Then, up to present, it is difficult to distinguish between IPL, fractional CO_2_ laser and RF from the molecular mechanisms of skin rejuvenation. Lastly, the original study of GSE39170 dataset used IPL for skin rejuvenation investigation. GSE39170 dataset was generated from IPL treatment investigation [11]. Meanwhile, GSE39170 dataset is employed in our current study for further research. Base on all above considerations, IPL treatment is employed in our study here.

Despite various skin rejuvenation approaches available presently, the exploration of causative factors associated with skin aging may delay or even prevent the occurrence and progression of skin aging. Previous studies revealed that dysregulation of PI3K was widely found in the development of different diseases [24, 25, 26], including skin aging [7, 27, 28, 29]. The major function of PI3K is to convert phosphatidylinositol (4,5)‐bisphosphate (PIP2) into phosphatidylinositol (3,4,5)‐trisphosphate (PIP3). On the cell membrane, protein kinase B (AKT) can bind to PIP3 via its pleckstrin homology (PH) domain. 3‐phosphoinositide‐dependent protein kinase‐1 (PDK1) and mammalian target of rapamycin complex 2 (mTORC2) respectively phosphorylate AKT at the Thr308 and Ser473 residues and thus completely activate AKT, which activates or inhibits a series of target genes, eventually regulating cell proliferation, apoptosis, survival, and protein synthesis [25, 30, 31]. It was reported that the activation of PI3K and AKT elevated MMP‐1 expression resulting in more collagen degradation in the skin [7, 32]. Inhibition of PDK1, a potential skin rejuvenation target, reverted cellular senescence in human dermal fibroblasts (HDFs) [8]. In higher eukaryotes, PI3K isoforms can be categorized into class I, II, and III according to the sequence homology and substrate preference [24]. Class I PI3K is the most characterized isoform of the PI3K family [24], which consists of a catalytic subunit (p110α, β, γ, and δ) and a regulatory subunit (p85α and β) that plays pivotal roles in cell proliferation, differentiation, and apoptosis. Phosphoinositide‐3‐kinase regulatory subunit 1 (PIK3R1), which encodes the predominant regulatory subunit p85α of class I PI3K, binds with p110 kinase to form a PI3K holoenzyme [24, 25]. As a tumor suppressor gene, the abnormal expression of PIK3R1 is associated with cell proliferation and apoptosis [24, 26]. There is increasing evidence revealing that PIK3R1 is linked to the pathophysiology of cancers [24, 25, 26]. Nonetheless, the precise role of PIK3R1 in skin aging and rejuvenation is still unknown. To sum up, we hypothesize that PIK3R1 might perform its function through PIP3 recruiting AKT on the cytoplasmic membrane and further activation of AKT by PDK1 and mTORC2, regulating transcription factors and/or target genes (FOXO1, Bcl‐2, and/or MMP‐1) to affect skin fibroblast senescence, proliferation, apoptosis, and collagen (I and III) production and degradation, eventually playing an important role in skin aging and rejuvenation.

Now that the pathophysiological mechanisms of PIK3R1 during the processes of skin aging and skin rejuvenation have not yet been investigated. Consequently, we sought to conduct a comprehensive bioinformatic analysis [33], molecular confirmation of PIK3R1 and function experiments based on gene expression data, functional annotations and biological validation, which might gain insight into mechanistic underpinnings of PIK3R1 in skin aging and rejuvenation.

## 2 Materials and Methods

### 2.1 Data Resources

Microarray RNA sequencing data from the left forearm of 15 human samples (5 aged untreated, 5 aged treated and 5 young samples) was accessible through Gene Expression Omnibus (GEO, RRID:SCR_005012) under the series accession number GSE39170 (https://www.ncbi.nlm.nih.gov/geo/query/acc.cgi?acc=GSE39170). Illumina Genome Analyzer II on the GPL9115 platform was used to detect the expression of 16 790 annotated genes with 31 700 probes. To reduce the prediction error for different samples, the normalization of gene expression profiles was processed with the *normalizeBetweenArrays* function in the *limma* (RRID: SCR_010943) package of R software version 3.6.2 [33].

### 2.2 Gene Set Enrichment Analysis (GSEA)

GSEA (RRID: SCR_003199) is a widely used computational method to assess whether functional enrichment of holistic predefined gene set is statistically significant between two biological states [33]. Utilizing *GSEABase ClusterProfler*, and *enrichplot* packages, enrichment scores of biological processes (BP) from gene ontology (GO) terms were estimated and sorted by setting the parameter to 1000 gene set permutations. A *p* value cutoff of 0.05 was selected for significant enrichment, with functional annotation results of GSEA visualized adopting *ggplot2* (RRID: SCR_014601) function [33].

### 2.3 Differential Expression Analysis

The *lmFit* and *eBayes* functions in *limma* packages were used to filtrate differentially expressed genes (DEGs) between young/aged untreated, aged untreated/aged treated, and young/aged treated skin. Analyses of two‐dimensional hierarchical clustering and volcano plot were conducted employing the *limma* package in R [33]. Threshold of statistical significance was defined as fold change (FC) ≥ 1.5 and a false discovery rate (FDR)‐ adjusted *p* < 0.05.

### 2.4 Coexpression Network Analysis

To investigate the PIK3R1 mechanisms in skin aging and rejuvenation, we conducted weight gene correlation network analysis (WGCNA) of overlapping DEGs among young/aged untreated, aged untreated/aged treated, and young/aged treated groups. WGCNA, a superiorly proven data excavation technology, can not only establish the unbiased coexpression network to convert bulk microarray data into gene coexpressed modules, but also endow these modules with biological functions that functionally correlate with genomic phenotypes and clinical traits [33]. First, the hclust (RRID: SCR_009154) function was employed to eliminate outlier samples with low inter‐array correlation, which could guarantee the dependability of the unsupervised signal network construction. Then, the optimal soft threshold (power = 16) was selected using the *pick‐SoftThreshold* function, an algorithm that endeavors to keep the signal network approximate to the authentic biological condition via refining integral connectivity of the coexpression modules [33]. Module eigengene (ME), representative of the entire expression levels of module genes, was computed by the *moduleEigengenes* function. Pearson correlation coefficient (PCC) was calculated to estimate the relation between ME and each gene in a module. Finally, a hierarchical clustering tree was constructed according to the values of PCC, in which functionally similar genes (*n* ≥ 30) were aggregated to form a branch (or coexpression module). Adopting *clusterProfiler* (RRID: SCR_016884) package in R, Kyoto Encyclopedia of Genes and Genomes (KEGG, RRID: SCR_012773) pathway analyses were performed to discern pathophysiological mechanisms that were most affected by predetermined DEGs [33].

### 2.5 Cross‐Talking Pathways of PIK3R1 and Signature Genes

Employing *verboseScatterplot* package, we depicted the scatter diagram of the correlation between module membership (MM) and gene significance (GS), which could separately reflect intramodular connectivity and phenotypic trait in WGCNA [33]. Among coexpressed modules, DEGs with specific biological functions and strongly related to PIK3R1 were selected based on empirical criteria of connectivity values (MM > 0.4) and genotypic trait (GS > 0.5). We then uploaded the set of DEGs into the online STRING (RRID: SCR_005223) database and thus to create a global regulatory network of protein–protein interaction (PPI) encoded by these DEGs. Lastly, the cross‐talking pathways of PIK3R1 in this network were enriched using KEGG analysis. Visualization of global regulatory network and cross‐talking signals was accomplished adopting *cytoscape* (RRID: SCR_003032) software [33].

Pearson correlation analysis was used to analyze the quantitative correlation between a given gene and pathway genes [34]. For each cross‐talk pathway, the top five genes with the highest PCC values were deemed as signature genes, which were representative of the closest relation to other genes in a pathway. In case that the pathway signature genes showed a significantly statistical relation to the given genes (e.g., PIK3R1), this pathway was regarded to be mediated or regulated via these given genes.

### 2.6 Analysis of Receiver Operating Characteristic Curve (ROC)

ROC analysis was conducted to estimate the classifier performance of sequential output, including sensitivity and specificity parameters, as measured by the area under the curve (AUC) [33]. Diagnostic performance of PIK3R1 to distinguish young subjects from aged untreated subjects was assessed using the *pROC* package in R software. An AUC value of 100% was for complete prediction and 50% for random selection. All *p* values were bilateral, and statistical significance at *p* < 0.05 was selected.

### 2.7 Subjects

Twenty‐two Chinese female subjects (Fitzpatrick II–IV), including 11 young subjects ≤ 30 years old and 11 old subjects ≥ 60 years old, were recruited from the out‐patient clinic in the Department of Dermatology, The First Hospital of China Medical University. All of the ≥ 60 years old subjects had pre‐ and post‐treatment biopsies of fresh tissue for RT‐PCR and western blot assay of the biochemical changes following treatment. The exclusion criteria were as follows: retinoid use within the previous 6 months, having a history of keloidal tendency or photosensitivity, having any other skin diseases or any systemic diseases, or being in varying stages of pregnancy and lactation. During the study period, the subjects were not permitted to receive any other cosmetic treatment.

### 2.8 IPL Treatment and Tissue Collection

The closed envelope technique for randomization was employed to determine which side of the face would be selected as aged treated group to receive the IPL treatment (Lumenis One, Lumenis Co., Santa Clara) with a wavelength range from 560 to 1200 nm. Cutoff filters of 560, 590, and 640 nm were used for four IPL sessions at 4–5 weeks intervals [9]. The energy fluency ranged from 15 to 17 J/cm^2^. A double or triple pulse mode, with a pulse width of 3.0–4.0 ms and a delay time of 30–40 ms, were utilized according to our experience [9]. Biopsies were collected at 4 weeks after the last IPL treatment from the area before tragus and immediately transferred to RNAlater (Qiagen, Valencia, CA) or tissue protein extraction reagent (Thermo Scientific, Waltham, MA) for further analysis. For the bias reduction, the strategy of investigators and subjects blinding was employed during the conduct and analysis of the study.

### 2.9 TaqMan Real‐Time RT‐PCR

Total RNA was isolated from young skin, aged untreated skin, and aged treated skin using the RNeasy Mini Kit (Qiagen, Germantown, MD) according to the manufacturer's specifications. Reverse transcription reactions were performed using High‐Capacity cDNA Reverse Transcription kits (Applied Biosystems, Foster City, CA) [35]. For each reaction, 200 ng RNA was added. PCR reaction was done in the system mixed with 0.5 μL 20 × TaqMan Gene Expression assays, 5 μL TaqMan Gene Expression Master Mix, 20 ng cDNA template, and 2.5 μL RNase‐free water based on our experience [35]. Results were obtained from the average measured in triplicate and normalized to a control gene GAPDH. Fold changes were generated by calculating 2^−ΔΔCt^ [35].

### 2.10 Western Blot Assay

Western blot assay was employed to determine the levels of PIK3R1, PDK1, phosphorylated PDK1, p16, collagen I and III in skin tissues and/or HDFs. Protein samples were isolated on 12% sodium dodecyl sulfate polyacrylamide gel electrophoresis gel and transferred to a polyvinylidene fluoride membrane. In 20 mM Tris–HCl (pH 7.4) containing 150 mM NaCl and 0.05% Tween 20, the 10% skim milk‐blocked membrane was washed with Tris–HCl (pH 7.4) containing 150 mM NaCl and 0.05% Tween 20. Then, the anti‐PI3K p85α antibody (1:1000, ab133595, RRID: AB_3662733, Abcam, Waltham, MA, USA), anti‐PDK1 antibody (1:1000, ab202468, RRID: AB_3677427, Abcam), anti‐PDPK1 (phospho S241) antibody (1:1000, ab131098, RRID: AB_11159760, Abcam), anti‐p16 ARC antibody (1:2000, ab51243, RRID: AB_2059963, Abcam), anti‐Collagen I antibody (1:2000, ab316222, RRID: AB_3697422, Abcam), anti‐Collagen III antibody (1:1000, ab184993, RRID: AB_2895112, Abcam), anti‐AKT3 + AKT2 + AKT1 antibody (1:2000, ab300473, RRID: AB_3717774, Abcam), Phospho‐Akt (Ser473) antibody (1:1000, 4058, RRID: AB_331168, Cell Signaling Technology), Phospho‐Akt (Thr308) antibody (1:1000, 4056, RRID: AB_331163, Cell Signaling Technology), FoxO1 antibody (1:2000, 2880, RRID: AB_3717778, Cell Signaling Technology), Phospho‐FoxO1 (Ser256) antibody (1:2000, 9461, RRID: AB_3717779, Cell Signaling Technology), anti‐MMP1 antibody (1:2000, ab318207, RRID: AB_3717780, Abcam), anti‐Bcl‐2 antibody (1:2000, ab182858, RRID: AB_2715467, Abcam) or anti‐GAPDH antibody (1:5000, ab181602, RRID: AB_2630358, Abcam) for 1 h at 37°C. The membranes were then incubated with horseradish peroxidase‐conjugated IgG (1:5000, goat anti‐rabbit IgG antibody, ab205718, RRID: AB_2819160, Abcam, Waltham, MA, USA) for 0.5 h at 37°C, and protein was detected with enhanced chemiluminescence reagents (ECL, Pierce, IL, USA). Density analysis was performed using the ImageJ software (version 1.54p).

### 2.11 Cell Culture

Immortalization of human dermal fibroblasts (HDFs, SV40 transfection) was obtained from Cellverse Bioscience Technology Co. Ltd. (iCell‐0051a, Shanghai, China). The cells were cultured in high‐glucose DMED medium supplemented with 10% fetal bovine serum and maintained in a humidified incubator at 37°C with 5% CO_2_.

### 2.12 Lentiviral Transduction

The lentiviruses packaging PIK3R1 shRNA were purchased from Shanghai Genechem Co. Ltd. Each of these sequences was cloned into the corresponding vector. Lentiviral packaging experiments were conducted using Lipofiter, as manufacturer described. PIK3R1 knockdown plasmid was co‐transfected with the packaging plasmids (pMD2G, psPAX2) into HDFs. The lentivirus was collected 48 h and 72 h after transfection. To generate stable PIK3R1 knockdown cell lines, HDFs were transduced with lentiviruses and then selected with puromycin (5.5 μg/mL, Biosharp) after 1 week of production. RNA sequences: shPIK3R1–1: 5′‐CGGTACAGCAAAGAATACATA‐3′, shPIK3R1–2: 5′‐TCCAGCCTCGGTTTCTATTTA‐3′, shNegative Control (shNC): 5′‐CAGCATTAAACCAGACCTTAT‐3′.

### 2.13 Ultraviolet A (UVA) Irradiation

Cells were washed by phosphate‐buffered saline (PBS) before being exposed to UVA irradiation (5 J/cm^2^) using the UV 801 KL equipment (Waldmann, Germany). During irradiation, cells were in a thin layer of cool PBS, and the distance between the cells and the lamp was 15 cm. After irradiation, PBS was replaced by fresh medium, and cells were re‐cultured for the subsequent experiments. UVA irradiation was conducted once a day for 8 consecutive days to complete the chronic skin photoaging model of HDFs induced by UVA.

### 2.14 Senescence‐Associated β‐Galactosidase Staining

For HDFs cultured in 6‐well plates, the cells were washed once with PBS after aspirating culture medium. Subsequently, 1 mL β‐Gal fixative was added, followed by fixation at room temperature for 15 min. The fixative was removed, and cells were washed three times with 1× PBS (3 min per wash). After aspirating the PBS, 1 mL β‐Gal staining working solution (Senescence‐Associated β‐galactosidase (SA‐β‐Gal) Stain Kit, G1580, Solarbio, Peking, China) was added to each well. The plate was incubated at 37°C for 12–24 h and sealed with parafilm to prevent evaporation. Cells were observed under a standard optical microscope. SA‐β‐Gal‐positive cells were visualized and photographed under a standard optical microscope. Three random fields per group were analyzed for quantitative assessment.

### 2.15 EdU Staining for Cell Proliferation Assay

E‐Click EdU Cell Proliferation Imaging Assay Kit (Red, Elab Fluor 594) (E‐CK‐A377, Elabscience, Wuhan, China) was used for cell proliferation detection according to the manufacturer's instruction. During the procedure, cells were washed with 500 μL PBS containing 3% BSA (ST2254, Beyotime Biotechnology, Shanghai, China). Permeabilization was performed using 500 μL PBS with 0.3% Triton X‐100 (P0096, Beyotime Biotechnology). After DNA counterstaining with DAPI solution, fluorescence microscopy (Olympus BX53, RRID:SCR_022568, Shinjuku, Tokyo, Japan) was performed using appropriate filters.

### 2.16 Apoptosis Analysis by Flow Cytometry

The number of apoptotic cells was quantified using an Annexin‐V Apoptosis Detection Kit PE (Cat#88‐8102‐72, RRID: AB_2575183, Thermo Scientific, Waltham, MA, USA) according to the manufacturer's instructions. Early apoptotic cells were defined as Annexin‐V‐positive and 7‐AAD‐negative cells. Analyses were performed using a BD LSRFortessa Cell Analyzer (RRID:SCR_018655, BD Biosciences, San Jose, CA, USA) and FlowJo version 10.0.7 software (RRID:SCR_008520, FlowJo LLC, Ashland, OR, USA).

### 2.17 Statistical Analysis

Both R (version 3.6.2) and GraphPad Prism (version 9.4.0, RRID: SCR_002798) software were employed for statistical analyses. Unpaired two‐tailed *t*‐test and one‐way analysis of variance (ANOVA) were used for two groups and multiple groups comparisons. Correlation of coexpressed modules with phenotypic traits in WGCNA was estimated by Pearson correlation analysis. The ROC analysis was conducted to assess the predictive utility of PIK3R1 in model performance, with the accuracy expressed by AUC values [33]. All *p* values were bilateral and statistical significance at *p* < 0.05 was selected. The detail of analysis result was stated in the corresponding figure legend.

## 3 Results

### 3.1 Differentially Expressed Genes

**FIGURE 1:**
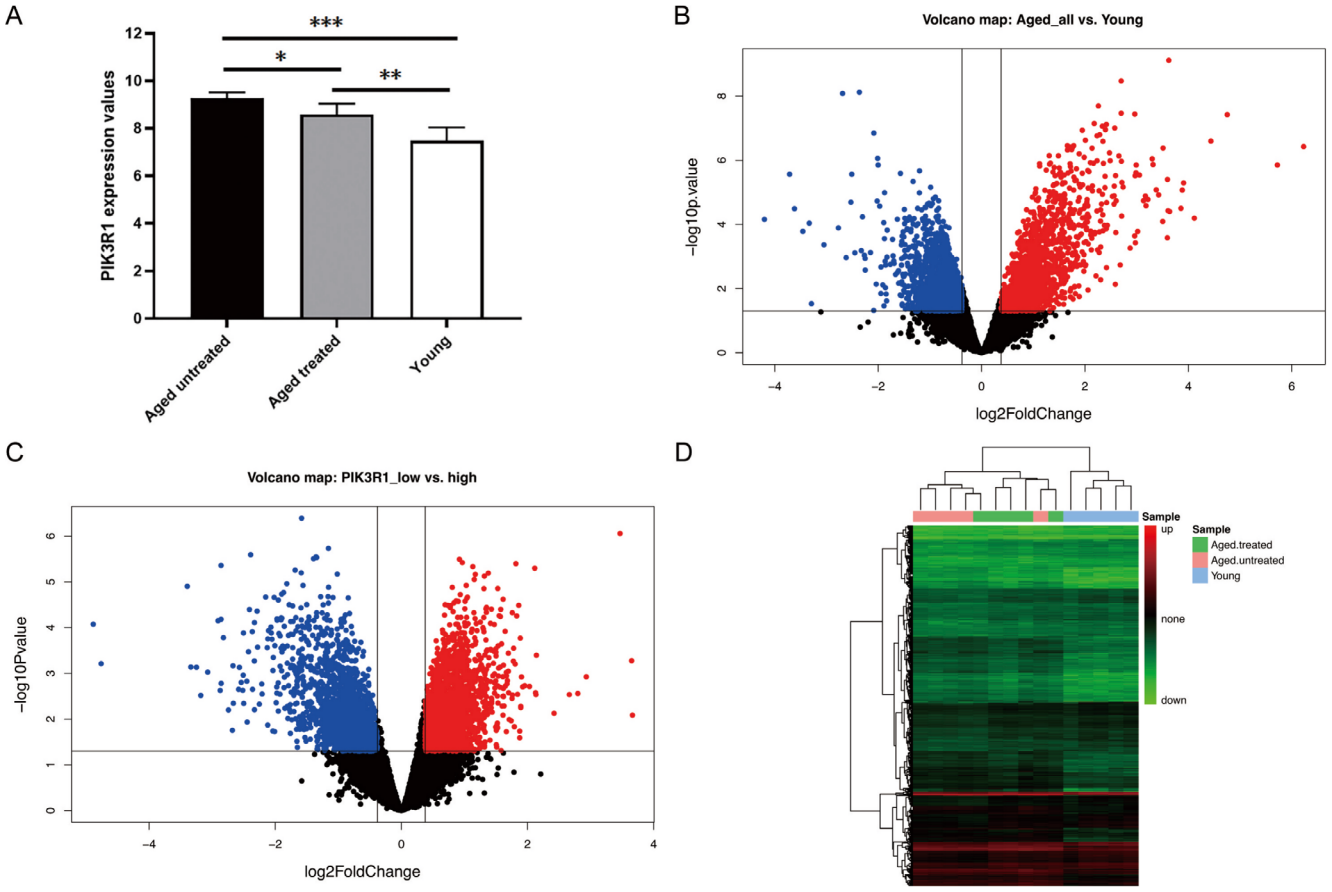
Gene expression and differentially expressed genes. (A) Comparison of PIK3R1 expression in aged untreated, aged treated and young groups. Aged untreated versus aged treated group (*n* = 5, *p* = 0.0201, *t* = 2.895, degree of freedom = 8, 95% confidence interval: −1.198 to −0.1356), aged treated versus young group (*n* = 5, *p* = 0.0084, *t* = 3.474, degree of freedom = 8, 95% confidence interval: −1.840 to −0.3719), aged untreated versus young group (*n* = 5, *p* = 0.0002, *t* = 6.526, degree of freedom = 8, 95% confidence interval: −2.400 to −1.147). Error bar indicated Mean ± SE. *p < 0.05, **p < 0.01, ***p < 0.001. Volcano plot of differentially expressed genes in aged/young (B) and PIK3R1‐low/high (C) groups: Red and blue represent up‐regulation and down‐regulation, respectively. (D) Heatmap of overlapping DEGs. DEGs, Differentially expressed genes. Green to red represents a change in gene expression from downregulated to upregulated.

The expression levels of PIK3R1 were the highest in aged untreated skin (9.26 ± 0.25), successively followed by aged treated skin (8.59 ± 0.45) and young skin (7.49 ± 0.55), which were statistically significant in pairwise comparison (*p* < 0.05, Figure 1A, Table S1). There was no significant difference of PDK1 expression among these three groups (Figure S1A). After removing redundant or unannotated probes, 11 964 background genes were retained for differential expression analysis. Of these, 3304 genes were expressed differentially in all aged samples versus young controls (Figure 1B), whilst 3330 DEGs were filtrated between PIK3R1‐low and high groups (Figure 1C). A total of 3271 DEGs (including 1685 down‐regulated and 1586 up‐regulated) were overlapped from PIK3R1‐low/high and aged/young groups. Holistic expression of overlapping DEGs for aged untreated, aged treated, and young skin was exhibited in the heatmap (Figure 1D).

### 3.2 Gene Co‐Expression Modules and Functional Annotation

**FIGURE 2:**
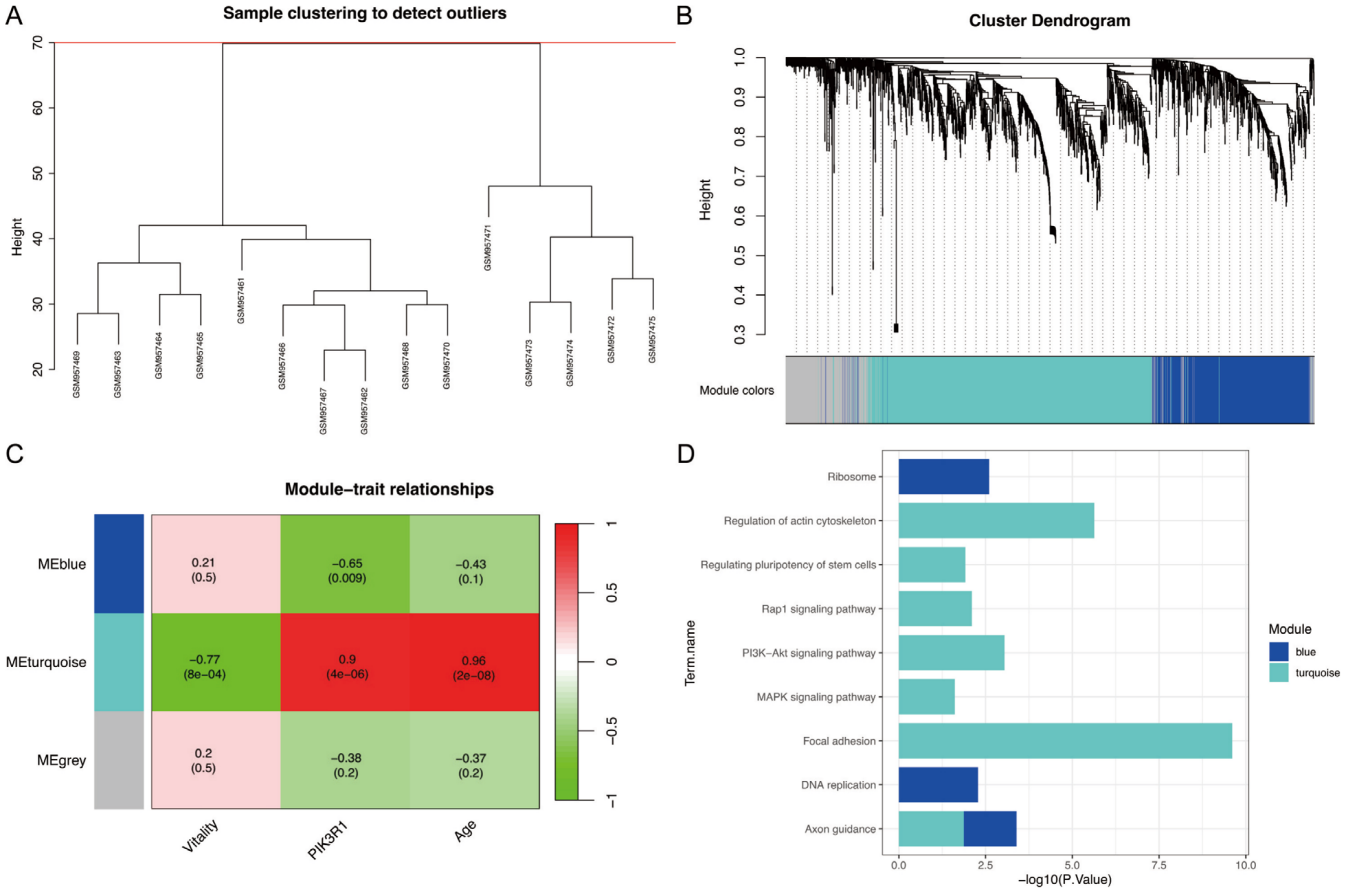
Clustering of samples and co‐expressed genes in WGCNA. (A) Sample clustering to discard outliers. (B) Cluster dendrogram of module genes assigned with different colors: Non‐clustering genes are grouped into gray module. (C) Relationships of co‐expression modules with phenotypes or traits in heatmap. (D) KEGG pathway analysis of module genes. KEGG, Kyoto Encyclopedia of Genes and Genomes; WGCNA, weight gene correlation network analysis.

During the process of WGCNA, all samples within the cut‐off line (Height = 70) were enrolled for hierarchical clustering (Figure 2A). To fit the scale‐free topology model, a soft‐thresholding power of 16 was chosen to construct the adjacency matrix. Co‐expression modules with unique colors were created and presented in the cluster dendrogram (Figure 2B). In the module‐trait relationship heatmap (Figure 2C), the turquoise module was significantly related to the phenotype of PIK3R1 (correlation coefficient (cor) = 0.9, *p* = 4e‐06) and the trait of vitality (cor = −0.77, *p* = 8e‐04), including age and rejuvenation processes. The blue module was not associated with vitality (cor = 0.21, *p* = 0.5), albeit with a negative correlation with PIK3R1 (cor = −0.65, *p* = 0.009).

Enrichment analyses of KEGG pathways (Figure 2D) were conducted to annotate co‐expression modules that were relevant to vitality and PIK3R1. We found that DEGs of the turquoise module were enriched in axon guidance, focal adhesion, regulating pluripotency of stem cells, regulation of actin cytoskeleton, MAPK, PI3K/AKT, and repressor/activator protein 1 (Rap1) signaling pathways. DEGs of the blue module were implicated in axon guidance, ribosome, and DNA replication.

### 3.3 Molecular Regulatory Network and PIK3R1‐Mediated Pathways

**FIGURE 3:**
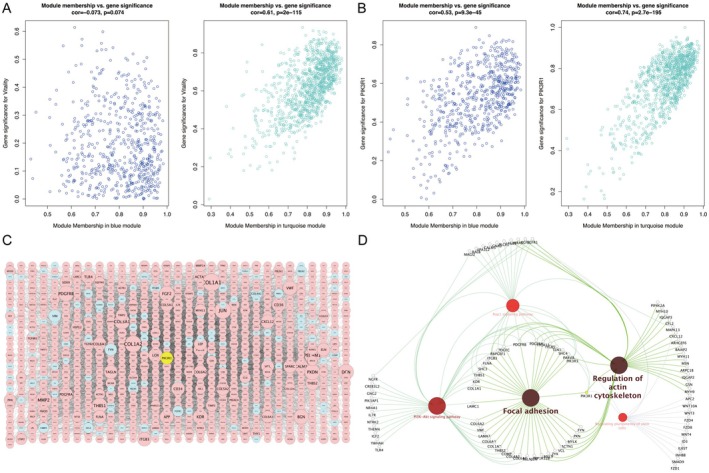
Molecular regulatory network and module‐pathway subnetwork in vitality. Scatterplots of module membership versus gene significance for vitality (A) and PIK3R1 (B). (C) Protein–protein interaction network of co‐expression module genes: Low expression of PIK3R1 is highlighted in yellow; red and blue represent up‐regulation and down‐regulation, respectively; larger node reflects higher gene connectivity. (D) Cross‐talking pathways of PIK3R1 in vitality.

Scatterplots reconfirmed the simultaneously significant relationship of turquoise module genes with vitality (cor = 0.61, *p* = 2e‐115, Figure 3A) and PIK3R1 (cor = 0.74, *p* = 2.7e‐195, Figure 3B) by measuring the correlation coefficient between module membership and gene significance. Thereafter, genes of turquoise module were extracted to establish a protein–protein interaction (PPI) network (Figure 3C). Molecular function of PIK3R1 was identified in module‐pathway subnetwork, which discerned the mechanistic pathway of PIK3R1 in vitality via focal adhesion, regulating pluripotency of stem cells, regulation of actin cytoskeleton, Rap1 and PI3K/AKT signaling pathways (Figure 3D, Table S2).

### 3.4 Mechanisms of PIK3R1 in Skin Aging and Rejuvenation

**FIGURE 4:**
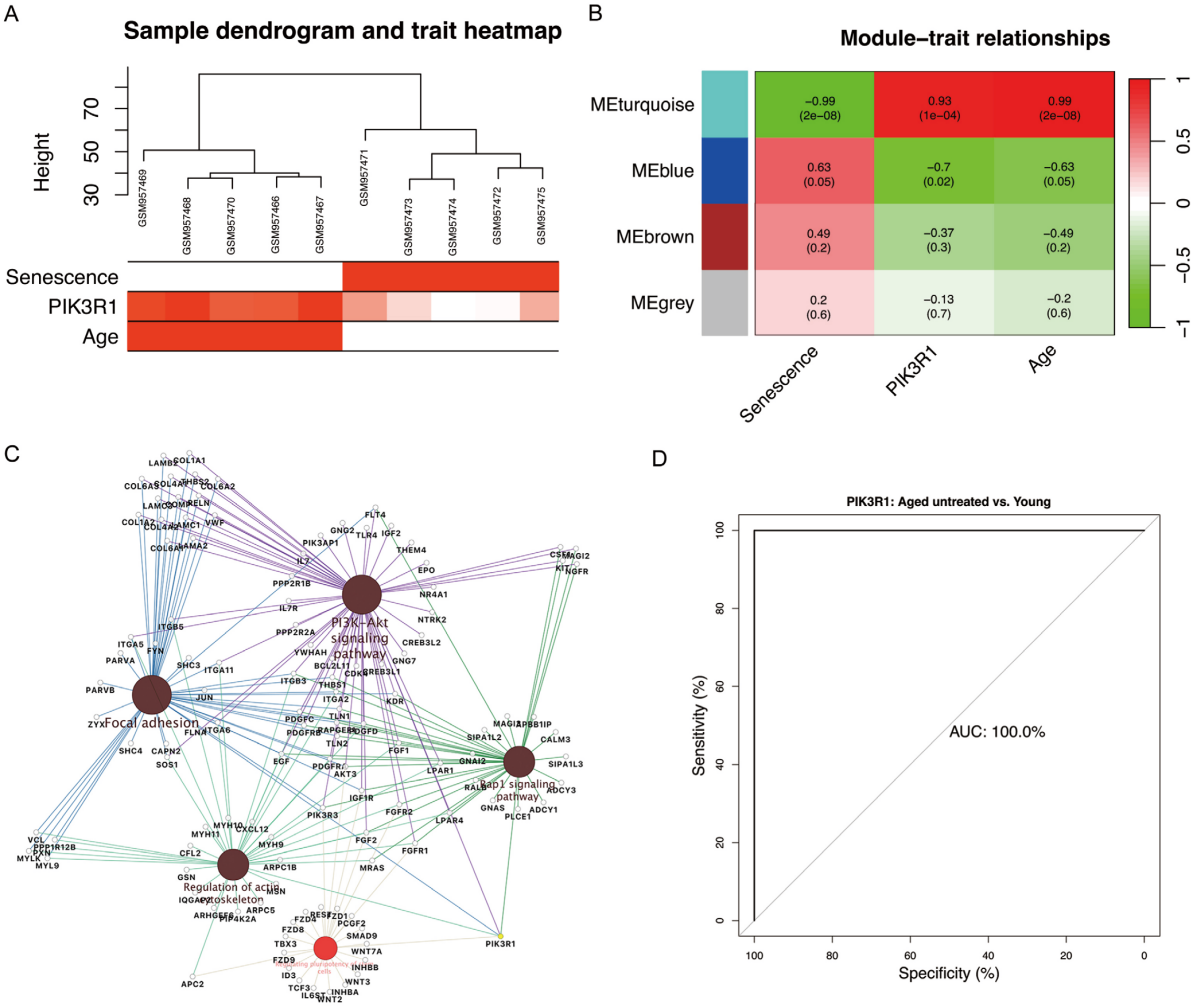
Functional identification of PIK3R1 in senescence. (A) Sample dendrogram and heatmap of senescence trait. (B) Relationships of co‐expression modules with senescence and PIK3R1. (C) Cross‐talking pathways of PIK3R1 in senescence. (D) ROC analysis of PIK3R1 in senescence. AUC, area under the curve; ROC, receiver operating curve.

Using DEGs between aged untreated and young groups, WGCNA was conducted to predict functional co‐expression modules associated with senescence (Figure 4A). Heatmaps of module‐trait relationships (Figure 4B) exhibited that the turquoise module had significant correlation with the trait of senescence (cor = −0.99, *p* = 2e‐08) and the phenotype of PIK3R1 (cor = 0.93, *p* = 1e‐04). DEGs of turquoise module were then profiled to construct the molecular regulatory network, wherein the mechanistic pathway of PIK3R1 in senescence was enriched in focal adhesion, regulating pluripotency of stem cells, regulation of actin cytoskeleton, Rap1, and PI3K/AKT signaling pathways (Figure 4C, detailed in Table S2). Based on the AUC values of 100% (Figure 4D), PIK3R1 possibly distinguished aged samples from young controls. Duo to the limitation of small sample size, the AUC of 100% might stem from overfitting. A further study with large sample size will be performed to verify this issue.

**FIGURE 5:**
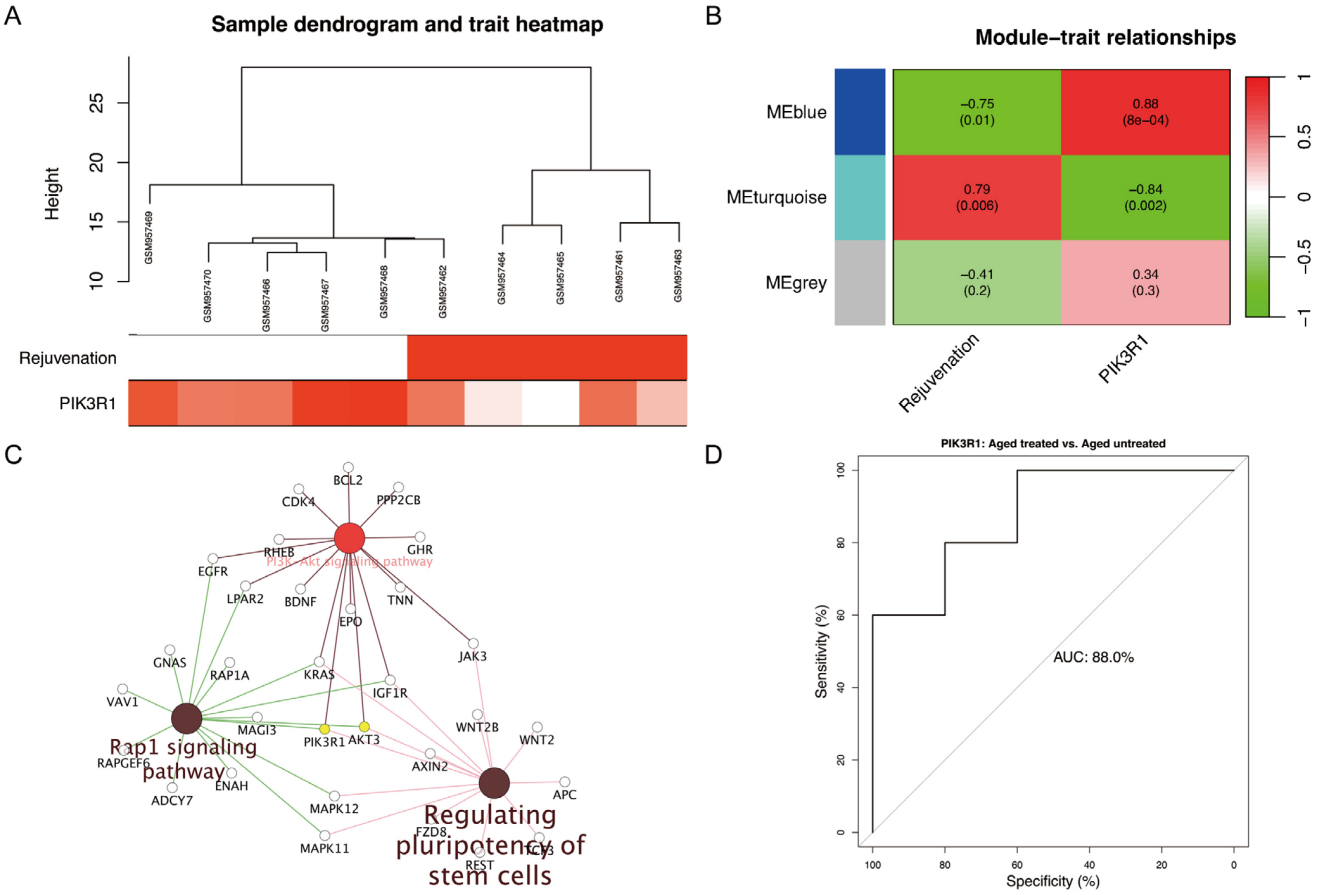
Functional identification of PIK3R1 in rejuvenation. (A) Sample dendrogram and heatmap of rejuvenation trait. (B) Relationships of co‐expression modules with rejuvenation and PIK3R1. (C) Cross‐talking pathways of PIK3R1 in rejuvenation. (D) ROC analysis of PIK3R1 in rejuvenation. AUC, area under the curve; ROC, receiver operating curve.

Likewise, we performed WGCNA to establish co‐expression modules associated with rejuvenation (Figure 5A) by employing DEGs between aged untreated and treated groups. Heatmaps of module‐trait relationships (Figure 5B) showed that blue and turquoise modules were statistically correlated with the trait of rejuvenation (blue: cor = −0.75, *p* = 0.01; turquoise: cor = 0.79, *p* = 0.006) and the phenotype of PIK3R1 (blue: cor = 0.88, *p* = 8e‐04; turquoise: cor = −0.84, *p* = 0.002). Thenceforth, we uploaded DEGs of blue and turquoise modules to build the molecular regulatory network, in which the mechanistic pathway of PIK3R1 in skin rejuvenation was involved in regulating pluripotency of stem cells, Rap1 and PI3K/AKT signaling pathways (Figure 5C, Table S2). According to the AUC value of 88% (Figure 5D), PIK3R1 could accurately differentiate between aged untreated and treated human skin transcriptomes. According to the PCC values, the signature genes in each of the cross‐talking pathways were identified (Table S3). The expressions of PIK3R1 were either positively or negatively interacting with the pathway signature genes (*p* < 0.05) (Figure S2).

### 3.5 Verification of PIK3R1 and PDK1 by Real‐Time RT‐PCR and Western Blot Assay

**FIGURE 6:**
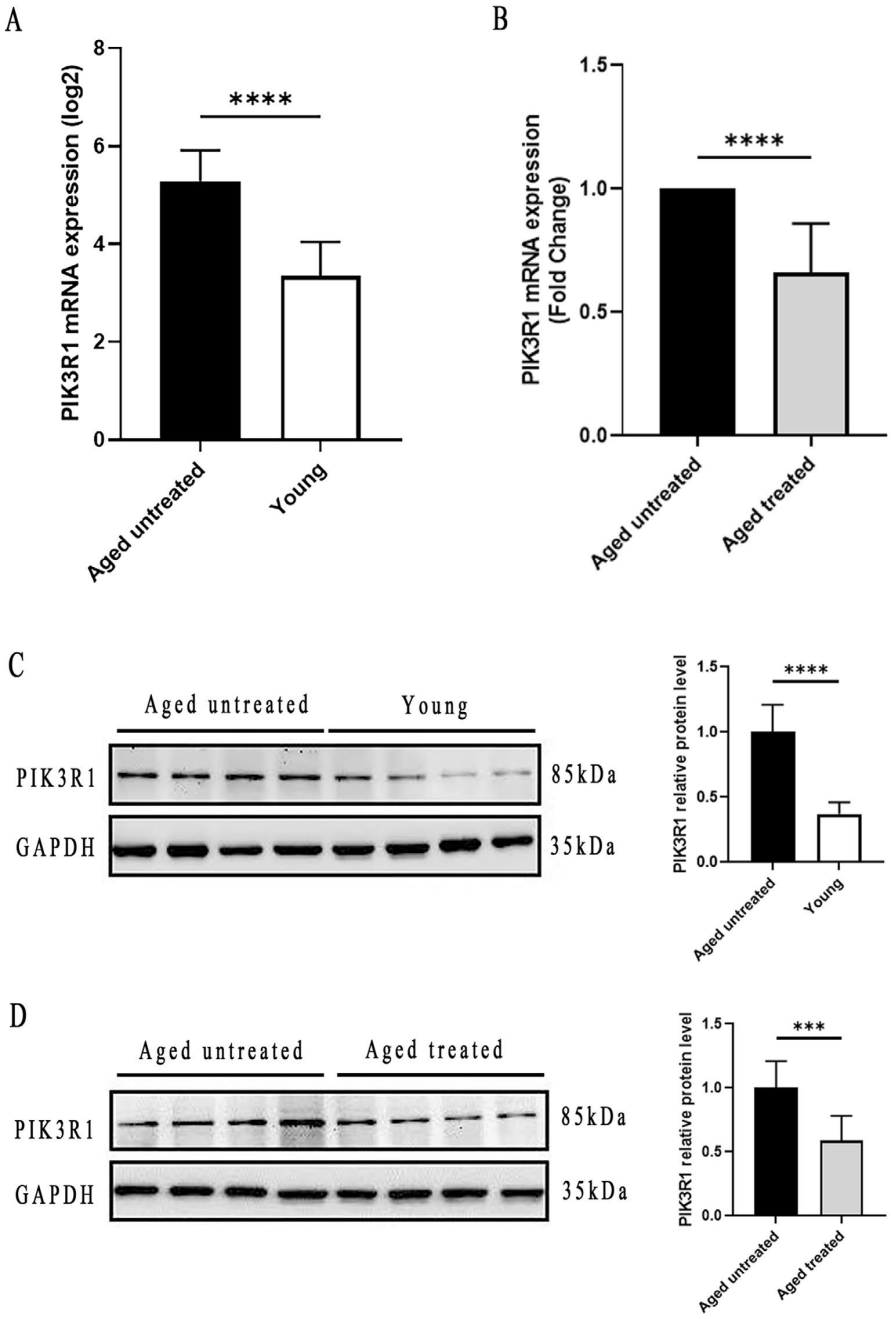
Detection of PIK3R1 mRNA and protein levels in different groups. (A, B) TaqMan Real‐time RT‐PCR showed that PIK3R1 mRNA expression levels were dramatically decreased in both young and aged treated groups, compared with the aged untreated group respectively. Aged untreated versus aged treated group (*n* = 11, fold change = 1.52, 95% confidence interval: −0.4709 to −0.2071). (C, D) Western blot assay revealed that PIK3R1 protein levels were significantly declined in both young and aged treated groups, compared with the aged untreated group respectively. Aged untreated versus young group (*n* = 11, fold change = 2.7, 95% confidence interval: −0.7737 to −0.4863). Aged untreated versus aged treated group (*n* = 11, fold change = 1.69, 95% confidence interval: −0.5881 to −0.2319). All results are either representative data or the Mean ± SE of the values obtained in three independent experiments. Error bar indicated Mean ± SE. ***p < 0.001, ****p < 0.0001.

IPL was proved to be an effective therapy in treating aging skin. After IPL treatment, the pigmented concerns, vascular problems, wrinkles, texture and elasticity of aged skin have been significantly improved (Figure S3). Skin tissue samples from 11 aged untreated subjects (≥ 60 years old, 7 females and 4 males, mean ± SD age = 66.27 ± 9.88 years) and 11 young subjects (≤ 30 years old, 7 females and 4 males, mean ± SD age = 23.18 ± 6.54 years) were collected for Real‐time RT‐PCR analysis and western blot assay. Skin tissue samples before and after IPL treatment from 10 aged subjects (≥ 60 years old, 6 females and 4 males, mean ± SD age = 66.80 ± 9.65 years) were employed for real‐time RT‐PCR analysis and western blot assay. As compared with that observed in young subjects and aged treated subjects, PIK3R1 mRNA expression was markedly upregulated in aged untreated subjects (Figure 6A,B). Result from western blot assay revealed that the expression of PIK3R1 protein was significantly decreased in young skin and aged treated skin compared with aged untreated skin respectively (Figure 6C,D). In young and aged treated groups, PDK1 mRNA and protein including phosphorylated PDK1 protein expression levels were not different from aged untreated group (Figure S1B–E).

### 3.6 Functional Analysis of PIK3R1 in Cell Senescence, Proliferation, Apoptosis and Collagen Expression

**FIGURE 7:**
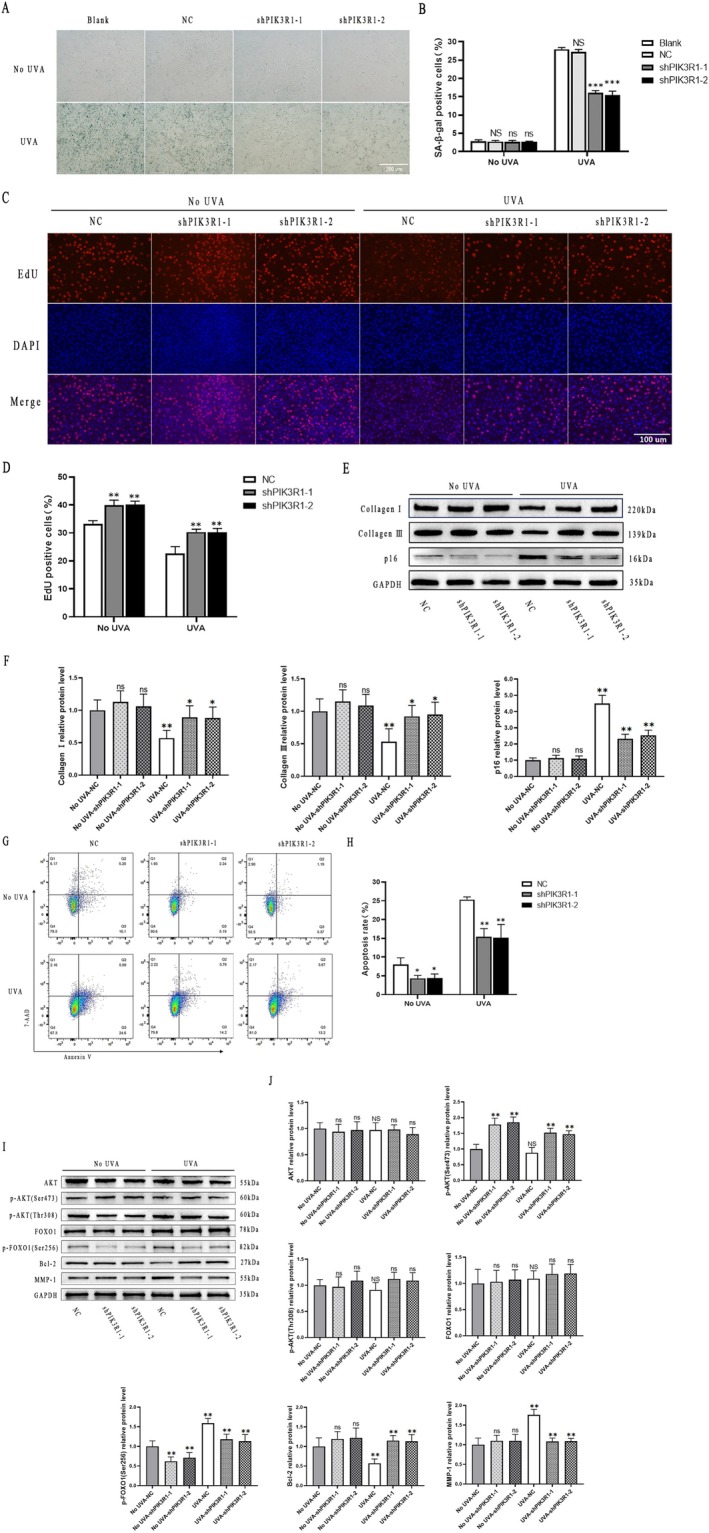
Knockdown of PIK3R1 in HDFs for cell senescence, proliferation, apoptosis, collagen (I and III) expression and potential targets detection. (A, B) β‐galactosidase staining showed that knockdown of PIK3R1 brought more resistance to UVA induced cell senescence in HDFs. Scale bars, 200 μm. NS, no significance compared with blank group. ns, no significance compared with NC group. ***p < 0.001 compared with NC group. (C, D) EdU staining showed that HDFs with PIK3R1 knockdown brought more proliferation in the no UVA treatment group and more resistance to UVA induced cell proliferation inhibition in the UVA treated group. Scale bars, 100 μm. **p < 0.01 compared with NC group. (E, F) Western blot assay showed that knockdown of PIK3R1 partly reversed collagen I and III expression decline caused by UVA in HDFs. PIK3R1 knockdown decreased the cellular senescence marker p16 in both no UVA and UVA treated groups. ns, no significance compared with NC group. Error bar indicated Mean ± SE. *p < 0.05, **p < 0.01. (G, H) 7‐AAD and Annexin V staining indicated that knockdown of PIK3R1 decreased HDFs apoptosis in the no UVA group and partly reversed HDFs apoptosis caused by UVA. Error bar indicated Mean ± SE. *p < 0.05, **p < 0.01. (I, J) Western blot assay showed that PIK3R1 knockdown increased p‐AKT (Ser473) and decreased p‐FOXO1 (Ser256) in both no UVA and UVA treated groups. Meanwhile, PIK3R1 knockdown increased Bcl‐2 and decreased MMP‐1 in UVA treated HDFs. NS, no significance compared with no UVA‐NC group. ns, no significance compared with NC groups respectively. Error bar indicated Mean ± SE. **p < 0.01. All results are either representative data or the Mean ± SE of the values obtained in three independent experiments.

Based on the alteration of PIK3R1 mRNA and protein in young and aged treated skin, we inferred that PIK3R1 might perform its function through regulating HDFs senescence, proliferation, apoptosis, and collagen amount in skin aging and rejuvenation. Knockdown of the PIK3R1 gene in HDFs by lentivirus and chronic skin photoaging by UVA irradiation were employed for further investigation. Compared with no UVA treated HDFs, UVA treatment dramatically upregulated SA‐β‐gal positive cells ratio both in blank and NC groups. With PIK3R1 knockdown, HDFs were more resistant to UVA induced cell senescence. In UVA treated group, the SA‐β‐gal positive cells ratios were 27.97 ± 0.46 in blank, 27.24 ± 0.65 in NC, 16.07 ± 0.63 in shPIK3R1‐1, and 15.46 ± 1.07 in shPIK3R1‐2. There was no statistical difference not only between blank and NC groups but also between shPIK3R1‐1 and shPIK3R1‐2 groups. The difference was statistically significant in shPIK3R1‐1 and shPIK3R1‐2 compared with NC. In the no UVA treatment group, there was no difference between PIK3R1 knockdown and NC samples (Figure 7A,B).

Cell proliferation was detected by EdU staining. The results indicated that UVA treatment significantly downregulated cell proliferation. Without UVA treatment, the EdU positive cell ratios were 33.31 ± 1.06 in NC, 39.84 ± 1.89 in shPIK3R1‐1, and 40.16 ± 1.21 in shPIK3R1‐2. The difference was statistically significant in shPIK3R1‐1 and shPIK3R1–2 compared with NC. It indicated that the knockdown of PIK3R1 resulted in more cell proliferation in the no UVA group. In UVA treatment group, the EdU positive cell ratios were 22.60 ± 2.52 in NC, 30.31 ± 1.08 in shPIK3R1‐1, and 30.34 ± 1.26 in shPIK3R1–2. The difference was statistically significant in shPIK3R1‐1 and shPIK3R1‐2 compared with NC (Figure 7C,D). It indicated that the knockdown of PIK3R1 resulted in more resistance to cell proliferation inhibition by UVA.

For cell senescence, p16 detection is also a method. We found that p16 expression was elevated after UVA treatment compared with the no UVA treated group. Both in the no UVA and UVA treatment groups, shPIK3R1 downregulated p16 expression levels. It showed that knockdown of PIK3R1 could decrease HDFs senescence. Western blot also revealed that collagen I and III expression levels reduced after UVA treatment. After knockdown of PIK3R1, collagen I and III expression levels were partly elevated in the UVA treatment group (Figure 7E,F).

7‐AAD and Annexin V double staining was employed for cell apoptosis detection. The results indicated that UVA treatment significantly upregulated the apoptosis ratio, from 8.06 ± 1.77 to 25.30 ± 0.70 in NC groups. In the no UVA treatment group, the ratio is 4.35 ± 0.77 (shPIK3R1‐1) and 4.39 ± 1.11 (shPIK3R1‐2). In UVA treatment group, the ratio is 15.43 ± 2.14 (shPIK3R1‐1) and 15.17 ± 3.49 (shPIK3R1‐2). Compared with corresponding NC groups, there were statistically different both the no UVA and UVA treated (*p* < 0.01) groups (Figure 7G,H). In indicate that PIK3R1 knockdown resulted in more resistant to cell apoptosis by UVA.

### 3.7 AKT, p‐AKT, FOXO1, p‐FOXO1, Bcl‐2 and MMP‐1 Expression Levels

To further investigate the molecular mechanism of PIK3R1 knockdown induced more resistance to cell senescence, proliferation inhibition, apoptosis and collagen synthesis decline caused by ultraviolet A irradiation in HDFs, we detected AKT, p‐AKT (Ser473), p‐AKT (Thr308), FOXO1, p‐FOXO1 (Ser256), Bcl‐2 and MMP‐1 expression levels. Compared with no UVA treated HDFs, UVA treatment increased p‐FOXO1 (Ser256), MMP‐1 levels and decreased Bcl‐2 levels (No UVA‐NC group vs. UVA‐NC group; Figure 7I,J). But AKT, p‐AKT (Ser473), p‐AKT (Thr308) and FOXO1 levels were not changed. In the no UVA group, PIK3R1 knockdown upregulated p‐AKT (Ser473) and downregulated p‐FOXO1 (Ser256) levels compared with NC group respectively. AKT, p‐AKT (Thr308), FOXO1, Bcl‐2 and MMP‐1 levels were not changed. In the UVA treated HDFs, PIK3R1 knockdown increased p‐AKT (Ser473) and Bcl‐2 levels, decreased p‐FOXO1 (Ser256) and MMP‐1 levels, compared with NC group respectively. Meanwhile, AKT, p‐AKT (Thr308) and FOXO1 levels were not altered (Figure 7I,J).

## 4 Discussion

To further improve our understanding of skin aging and rejuvenation modified by PIK3R1, we took advantage of RNA sequencing data to screen differentially expressed genes between aged untreated skin and young skin, aged untreated skin and aged treated skin for integrative genomic analysis. The findings emerging from WGCNA suggested that DEGs of co‐expression modules were significantly correlated with skin aging, skin rejuvenation, and PIK3R1 expression, which were enriched in PI3K/AKT, regulating pluripotency of stem cells, Rap1 signaling pathway, etc.

Previous evidence has indicated that the PI3K/AKT signaling pathway was involved in skin aging [7, 8, 32, 36]. As known, reactive oxygen species (ROS) play an important role in skin aging. Noh et al. proved that ROS in replicative aged HDFs was generated by the modulation of PIP3 metabolism [36]. As a lipid phosphatase, phosphatase and tensin homolog (PTEN) transforms PIP3 into PIP2 and negatively regulates the PI3K/AKT pathway [37]. The decrease in PTEN protein is responsible for maintaining high levels of PIP3 in replicative aged HDFs and consequently mediates the activation of the PI3K/AKT pathway, which leads to protein kinase C ζ (PKCζ) activation and in turn increases ROS production through overexpression of nicotinamide adenine dinucleotide phosphate (NADPH) expression [36]. ROS can further repress collagen production and increase MMPs transcription resulting in collagen amount reduction [27]. Our study found that HDFs with PIK3R1 knockdown were more resistant to UVA‐induced cell senescence, proliferation inhibition, apoptosis, and collagen synthesis decline. Meanwhile, we also found that there was upregulation of p‐AKT (Ser473) and Bcl‐2, downregulation of p‐FOXO1 (Ser256), and MMP‐1 in PIK3R1 knockdown HDFs with UVA irradiation. It indicated that PIK3R1 and downstream targets should be involved in skin aging and rejuvenation. Type I collagen is the most abundant protein in the ECM, and the decrease of the type I collagen content is closely associated with degenerative changes of tissues as seen in the aged skin. Park et al. showed that esculetin enhanced type I procollagen expression by the activation of MAPK (ERK1/2, p38, and JNK) and PI3K/AKT pathways and probably through increasing specificity protein 1 (Sp1) transcription factor expression in HDFs, thus exerting a possible skin rejuvenation effect [38]. Coincidentally, propolis exhibited a potential skin rejuvenation effect through suppressing UV‐induced MMP‐1 production and blocked collagen degradation through directly suppressing PI3K activity and phosphorylation of PDK1 and AKT [7]. Our study was also consistent with these findings because our results indicated that PIK3R1 knockdown partly restored collagen I and III expression decline by UVA. Cellular senescence is a key hallmark of aging, which presents a state of permanent growth arrest in response to different stimuli such as telomere attrition, oxidative stress, DNA damage, and oncogenic stress [8]. Senescence is considered a dynamic multistep process that is reversible in certain specific situations. An et al. identified PDK1 as a potential target to convert the senescent state to the quiescent state based on the molecular regulatory network of cellular senescence and phosphoprotein array experiments in HDFs [8]. They also proved that the inhibition of PDK1 in senescent HDFs eradicated senescence hallmarks (senescence‐associated β‐galactosidase activity and abundant p53 binding protein 1) and restored entry into the cell cycle by suppressing both nuclear factor κB and the mTOR pathway, thus restoring skin regeneration capacity [8, 39]. Here we found that PDK1 mRNA expression, PDK1 and phosphorylated PDK1 protein expression levels in young and aged treated skin were not different from aged untreated skin. It might be caused by different genetic backgrounds, specimen sources (skin tissue, primary cell, or cell lines), experimental models, and detection methods from previous studies [7, 8, 39]. It is a pity that the PDK1 activity result is absent in the current study. Thus, the function of PDK1 can't be excluded yet. Although the activity of PDK1 can be influenced by subcellular localization, protein interactions, or other post‐translational modifications, our future study will still focus on this issue. Recently, Zou et al. performed a single‐cell transcriptomic analysis of human skin from different age donors and identified cell‐type‐specific aging associated genes [28]. Despite the lack of a detailed mechanism explanation, it indicated that PIK3R1 was increased in fibroblasts from the old subjects [28]. To some extent, this result also supports our current study. All of the above suggests that PIK3R1 might play an important role in skin aging and rejuvenation through the PI3K/AKT signaling pathway. We infer that decreased PIK3R1 potentially performs its function through partial activation of AKT and regulation of possible downstream targets (FOXO, Bcl‐2, and MMP‐1) [7, 30, 40] resulting in a decline of cellular senescence, apoptosis, collagen degradation, and elevation of cell proliferation and collagen production. In our future study, we will work on a more detailed mechanism on this pathway.

In addition to PI3K/AKT, our analysis also demonstrated the involvement of PIK3R1 in skin aging and rejuvenation through the Rap1 signaling pathway and regulating pluripotency of stem cells. Telomere shortening has been implicated in cellular senescence, which may cause certain aging phenotypes [41, 42]. It is widely known that UV radiation leads to excessive ROS production, causing telomere mutations and further cell senescence or death. As part of a six‐member protein complex named shelterin, Rap1 contributes to the maintenance of genome stability by protecting telomeric DNA ends from non‐homologous end joining and from homologous recombination that can change telomere length. Swanson et al. reported that as HDFs aged and their telomeres became shorter, the level of Rap1 decreased [43]. Recently, Lototska et al. also found that Rap1 specifically protected the telomeres of senescent cells from DNA damage [44]. Stock et al. suggested that aberrant expression and localization of the Rap1 shelterin protein contributed to age‐related phenotypes [45]. Consistent with our findings, the above evidence also suggested that the Rap1 signaling pathway might play important roles in skin aging potentially through telomere length modification.

A key hallmark to aging is the dysregulation or exhaustion of the endogenous stem cell population [46]. Recently, Liu and colleagues reported that genomic/oxidative stress‐induced differential expression levels of the hemidesmosome component collagen XVII (COL17A1) in individual stem cells generated a driving force for cell competition [47]. Stem cells with higher expression of COL17A1 were selected for homeostasis, but their eventual loss of COL17A1 limited their competition, thereby resulting in skin aging. The resultant hemidesmosome fragility and stem cell delamination depleted adjacent melanocytes and fibroblasts to promote skin aging. Moreover, the forced maintenance of COL17A1 rescued skin aging [47, 48, 49]. Meanwhile, there is other evidence pointing to a linkage between skin stem cells and skin aging [50, 51, 52]. Previous studies also found that the PI3K/AKT pathway played an important role in inhibiting senescence and promoting self‐renewal of human skin‐derived precursors in vitro [53, 54]. Up to the present, the role of PIK3R1 in skin aging through regulating skin stem cells has not been investigated. We hypothesize that PIK3R1 might regulate pluripotency of stem cells possibly through the PI3K/AKT axis thus participate in skin aging and rejuvenation, which might shed new light on the study of skin aging and rejuvenation.

Further Pearson correlation analysis demonstrated that PIK3R1 was significantly correlated with the signature genes of each cross‐talking pathway. These data provided computational statistical support for the mediated or regulated role of PIK3R1 in PI3K/AKT, regulating pluripotency of stem cells, and Rap1 signaling pathways related to skin aging and rejuvenation. According to the AUC of 100% and 88%, high PIK3R1 exhibited possibly excellent diagnostic performance in skin aging and rejuvenation, indicating that PIK3R1 was capable of being a robust predictor of skin vitality. It is not ignored that the AUC of 100% might stem from overfitting due to small sample sizes (*n* = 5 per group). A large‐sample study will be carried out to further confirm this issue.

## 5 Conclusion

In summary, our study preliminarily suggested that bioinformatic analysis was a promising approach to uncover the potentially pleiotropic roles of PIK3R1 in skin aging and rejuvenation. Meanwhile, RT‐PCR and western blot confirmed PIK3R1 mRNA and protein expression alteration. Functional experiments further revealed that HDFs with PIK3R1 knockdown represented less cell senescence, proliferation inhibition, apoptosis, and collagen I and III expression decline caused by UVA irradiation. Molecular mechanism investigation further indicated that AKT, FOXO1, Bcl‐2, and MMP‐1 might be responsible. These processes were possibly mediated through the PI3K/AKT, Rap1 signaling pathway, and regulating pluripotency of stem cells. Small sample size is the limitation in both bioinformatics analysis (*n* = 5 per group) and validation experiments (*n* = 11 per group). Thus, our study conclusion is preliminary. In vivo and in vitro biological experiments with large sample size are scheduled in the future to specify the underlying mechanisms identified in the current study.

## Author Contributions

**Zhike Zhou:** conceptualization, methodology, data curation, formal analysis, visualization, investigation, writing – original draft, writing – review and editing. **Sha Sha:** conceptualization, methodology, data curation, formal analysis, writing – original draft, writing – review and editing. **Xiangnan Zhou:** methodology, data curation, formal analysis, visualization. **Chundi He:** investigation, resources, writing – original draft, writing – review and editing. **Ting Xiao:** supervision, resources, writing – original draft, writing – review and editing. **Hong‐Duo Chen:** supervision, resources, writing – original draft, writing – review and editing. **Yan Wu:** data curation, formal analysis, supervision, writing – original draft, writing – review and editing. **Fenqin Chen:** conceptualization, methodology, data curation, formal analysis, supervision, writing – original draft, writing – review and editing. **Le Qu:** conceptualization, funding acquisition, methodology, supervision, data curation, formal analysis, visualization, investigation, writing – original draft, writing – review and editing.

## Funding

This study was supported by the Natural Science Foundation of Liaoning Province, China (Number 2022‐MS‐211).

## Ethics Statement

This study was approved by the Institutional Research Ethics Committee of The First Hospital of China Medical University and conducted in accordance with the Declaration of Helsinki.

## Consent

Informed consent was obtained from each subject in the current study.

## Conflicts of Interest

The authors declare no conflicts of interest.

## Supporting information


**Figure S1:** fsb271466‐sup‐0001‐FigureS1.pdf.

**Figure S2:** fsb271466‐sup‐0002‐FigureS2.pdf.

**Figure S3:** fsb271466‐sup‐0003‐FigureS3.pdf.

**Table S1:** fsb271466‐sup‐0004‐TableS1.pdf.

**Table S2:** fsb271466‐sup‐0005‐TableS2.pdf.

**Table S3:** fsb271466‐sup‐0006‐TableS3.pdf.

**Data S1:** fsb271466‐sup‐0007‐DataS1.pdf.

## Data Availability

Data will be obtainable from the authors on request. GSE39170 data is publicly available in the GEO database (https://www.ncbi.nlm.nih.gov/gds/?term=GSE39170).
