# Supplementary material for: Using an Unbiased Coexpression Network to Reveal Cross‐Talking Pathways of Phosphoinositide‐3‐Kinase Regulatory Subunit 1 in Skin Aging and Rejuvenation
Source: FASEB J. 2026 Jan 16;40(2):e71466. doi: 10.1096/fj.202402347RRRR (PMC12811739; doi:10.1096/fj.202402347RRRR)
Supplement: Supplementary file 1 — Figure S1: fsb271466‐sup‐0001‐FigureS1.pdf. [file FSB2-40-e71466-s002.pdf]

Supplementary Figure S1 Comparison of PDK1 expression in aged untreated, aged treated and young groups.

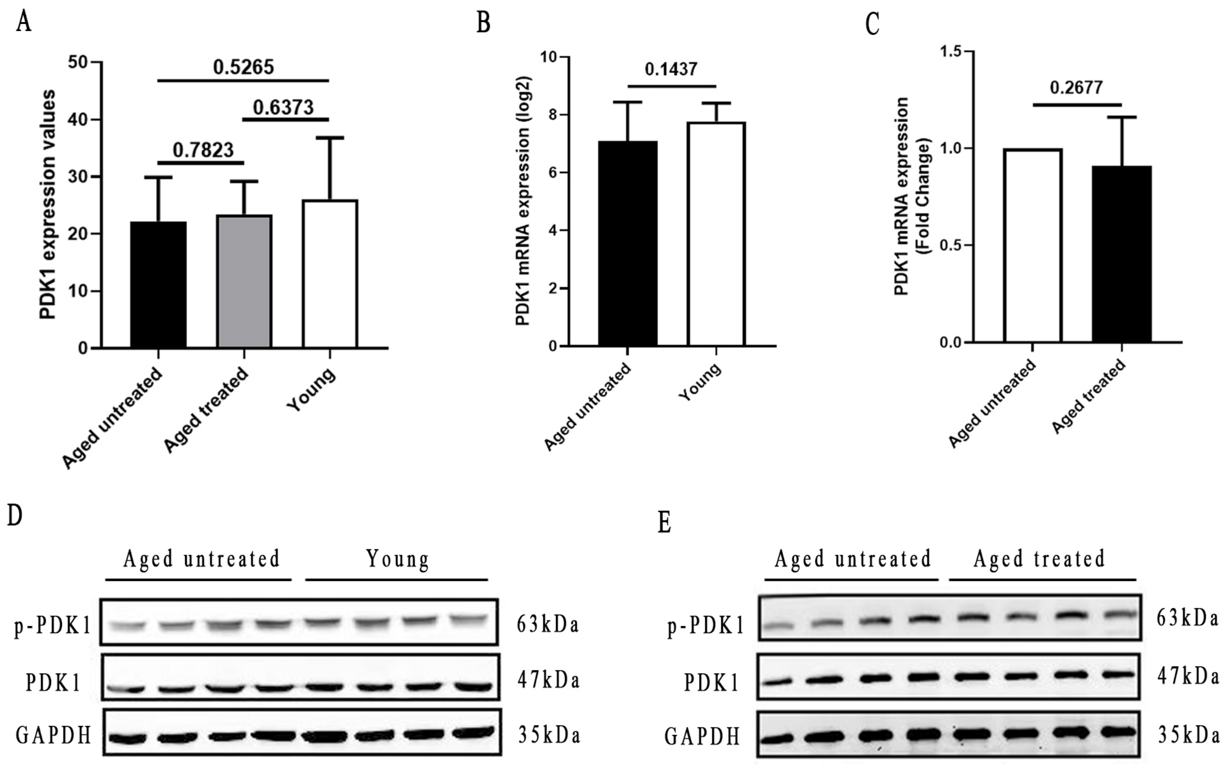

Figure S1 Comparison of PDK1 expression in aged untreated, aged treated and young groups. (A) Bioinformatics analysis indicated that there was no change of PDK1 expression in three groups. Aged untreated versus aged treated group (n = 5, P = 0.7823, t = -0.287, degree of freedom = 7.3852, 95% confidence interval: -11.291 to 8.827), aged treated versus young group (n = 5, P = 0.6373, t = -0.496, degree of freedom = 6.1236, 95% confidence interval: -15.906 to 10.524), aged untreated versus young group (n = 5, P = 0.5265, t = -0.665, degree of freedom = 7.2761, 95% confidence interval: -17.763 to 9.916). Error bar indicated Mean ± SE. ns indicated no significance. (B, C) Real-time RT-PCR showed that PDK1 mRNA expression levels were not changed in both young and aged treated groups, compared with aged untreated group respectively. Error bar indicated Mean ± SE. (D, E) Western blot assay revealed that PDK1 and phosphorylated PDK1 protein levels were not changed in both young and aged treated groups, compared with aged untreated group respectively.
