# Supplementary figures and images for: Using an Unbiased Coexpression Network to Reveal Cross‐Talking Pathways of Phosphoinositide‐3‐Kinase Regulatory Subunit 1 in Skin Aging and Rejuvenation
Source: FASEB J. 2026 Jan 16;40(2):e71466. doi: 10.1096/fj.202402347RRRR (PMC12811739; doi:10.1096/fj.202402347RRRR)

**Supplementary Figure S2** Quantitative correlation between PIK3R1 and signature genes.

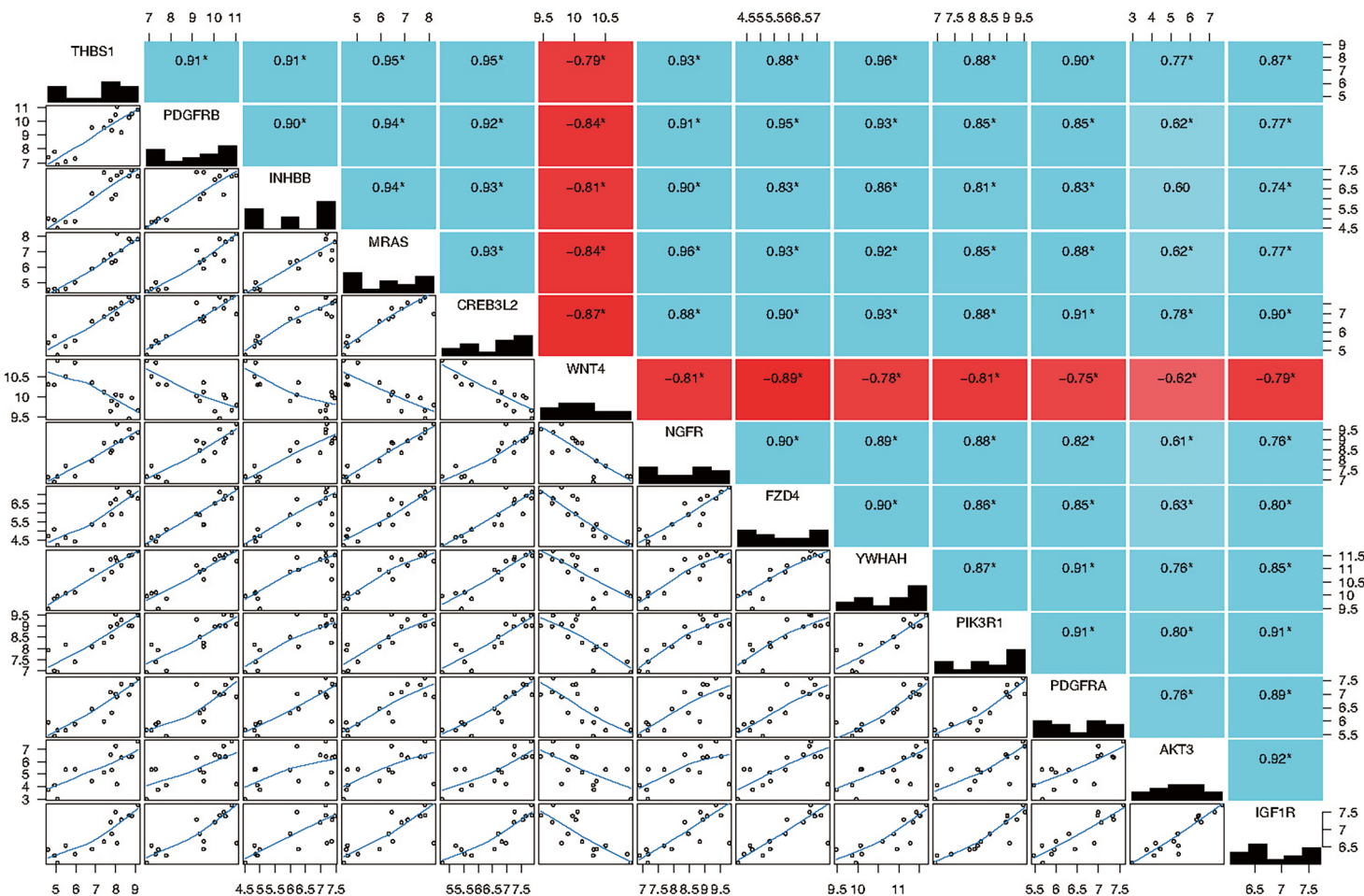

Supplement: Supplementary file 2 — Figure S2: fsb271466‐sup‐0002‐FigureS2.pdf. [file FSB2-40-e71466-s001.pdf]
