# Supplementary material for: Using an Unbiased Coexpression Network to Reveal Cross‐Talking Pathways of Phosphoinositide‐3‐Kinase Regulatory Subunit 1 in Skin Aging and Rejuvenation
Source: FASEB J. 2026 Jan 16;40(2):e71466. doi: 10.1096/fj.202402347RRRR (PMC12811739; doi:10.1096/fj.202402347RRRR)

Supplementary Figure S3 Clinical photos representing before (A) and after (B) IPL treatment from a 63-year-old subject.

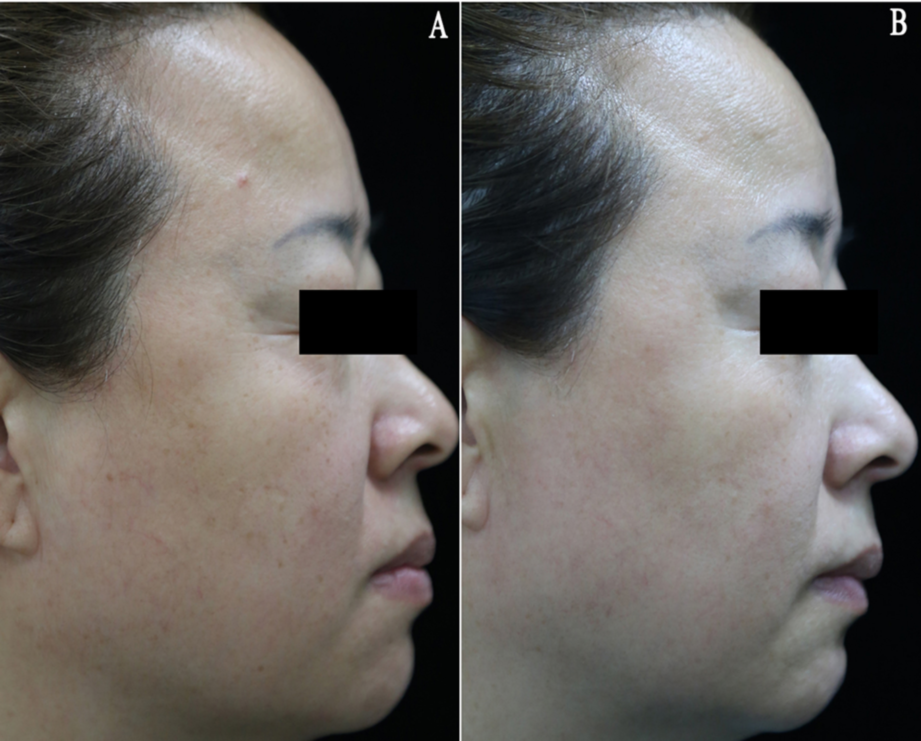

Supplement: Supplementary file 3 — Figure S3: fsb271466‐sup‐0003‐FigureS3.pdf. [file FSB2-40-e71466-s005.pdf]
