# Supplementary material for: Using an Unbiased Coexpression Network to Reveal Cross‐Talking Pathways of Phosphoinositide‐3‐Kinase Regulatory Subunit 1 in Skin Aging and Rejuvenation
Source: FASEB J. 2026 Jan 16;40(2):e71466. doi: 10.1096/fj.202402347RRRR (PMC12811739; doi:10.1096/fj.202402347RRRR)
Supplement: Supplementary file 4 — Table S1: fsb271466‐sup‐0004‐TableS1.pdf. [file FSB2-40-e71466-s006.pdf]

Supplementary Table S1 Clinical and phenotypic data of samples.

Sample	Group	PIK3R1	Age
GSM957466	Aged untreated	9.017912865	>=50
GSM957467	Aged untreated	9.475388031	>=50
GSM957468	Aged untreated	9.526234524	>=50
GSM957469	Aged untreated	9.291346743	>=50
GSM957470	Aged untreated	8.980614242	>=50
GSM957461	Aged treated	9.087704854	>=50
GSM957462	Aged treated	9.015997117	>=50
GSM957463	Aged treated	8.518853398	>=50
GSM957464	Aged treated	8.253919802	>=50
GSM957465	Aged treated	8.080896255	>=50
GSM957471	Young	8.168221592	<=30
GSM957472	Young	6.993592223	<=30
GSM957473	Young	7.403031358	<=30
GSM957474	Young	6.930576725	<=30
GSM957475	Young	7.930978013	<=30
