# Supplementary material for: Using an Unbiased Coexpression Network to Reveal Cross‐Talking Pathways of Phosphoinositide‐3‐Kinase Regulatory Subunit 1 in Skin Aging and Rejuvenation
Source: FASEB J. 2026 Jan 16;40(2):e71466. doi: 10.1096/fj.202402347RRRR (PMC12811739; doi:10.1096/fj.202402347RRRR)
Supplement: Supplementary file 5 — Table S2: fsb271466‐sup‐0005‐TableS2.pdf. [file FSB2-40-e71466-s004.pdf]

Supplementary Table S2 KEGG enrichment analysis of PIK3R1 in co-expression modules of the three traits.

ID	Description	GeneRatio	*P* value
**Aged all vs. Young**			
KEGG:04510	Focal adhesion	38/522	2.45E-10
KEGG:04810	Regulation of actin cytoskeleton	33/522	2.33E-06
KEGG:04151	PI3K-Akt signaling pathway	37/522	9.98E-04
KEGG:04015	Rap1 signaling pathway	22/522	7.85E-03
KEGG:04550	Regulating pluripotency of stem cells	16/522	1.20E-02
**Aged untreated vs. Young**			
KEGG:04510	Focal adhesion	52/827	1.54E-11
KEGG:04151	PI3K-Akt signaling pathway	63/827	1.07E-06
KEGG:04810	Regulation of actin cytoskeleton	41/827	5.23E-05
KEGG:04015	Rap1 signaling pathway	38/827	7.72E-05
KEGG:04550	Regulating pluripotency of stem cells	24/827	4.32E-03
**Aged treated vs. Aged untreated**			
KEGG:04550	Regulating pluripotency of stem cells	14/216	1.23E-05
KEGG:04015	Rap1 signaling pathway	15/216	2.35E-04
KEGG:04151	PI3K-Akt signaling pathway	16/216	1.80E-02

KEGG: Kyoto Encyclopedia of Genes and Genomes.
