# Supplementary material for: Using an Unbiased Coexpression Network to Reveal Cross‐Talking Pathways of Phosphoinositide‐3‐Kinase Regulatory Subunit 1 in Skin Aging and Rejuvenation
Source: FASEB J. 2026 Jan 16;40(2):e71466. doi: 10.1096/fj.202402347RRRR (PMC12811739; doi:10.1096/fj.202402347RRRR)
Supplement: Supplementary file 6 — Table S3: fsb271466‐sup‐0006‐TableS3.pdf. [file FSB2-40-e71466-s007.pdf]

Supplementary Table S3 Selection of signature genes from the cross-talking pathways.

Pathway	Genes	Signature genes
PI3K/AKT signaling pathway	AKT3, BCL2, BCL2L11, BDNF, CDK4, COL1A1, COL1A2, COL4A1, COL4A2, COL6A1, COL6A2, COL6A3, COMP, CREB3L1, CREB3L2, CSF1, EGF, EGFR, EPO, FGF1, FGF2, FGFR1, FGFR2, FLT4, GHR, GNG2, GNG7, IGF1R, IGF2, IL7, IL7R, ITGA11, ITGA2, ITGA5, ITGA6, ITGB3, ITGB5, JAK3, KDR, KIT, KRAS, LAMA2, LAMB2, LAMC1, LAMC3, LPAR1, LPAR2, LPAR4, MAGI2, NGFR, NGFR, NR4A1, NTRK2, PDGFC, PDGFD, PDGFRA, PDGFRB, PIK3AP1, PIK3R1, PIK3R3, PPP2CB, PPP2R1B, PPP2R2A, RELN, RHEB, SOS1, THBS1, THBS2, THEM4, TLR4, TNN, VWF, YWHAH	CREB3L2, THBS1, YWHAH, PDGFRB, PIK3R1
Rap1 signaling pathway	ADCY1, ADCY3, ADCY7, AKT3, APBB1IP, CALM3, CSF1, EGF, EGFR, ENAH, FGF1, FGF2, FGFR1, FGFR2, FLT4, GNAI2, GNAS, IGF1R, ITGB3, KDR, KIT, KRAS, LPAR1, LPAR2, LPAR4, MAGI2, MAGI3, MAPK11, MAPK12, MAPK13, MRAS, NGFR, PDGFC, PDGFD, PDGFRA, PDGFRB, PIK3R1, PIK3R3, PLCE1, RALB, RAP1A, RAPGEF1, RAPGEF6, SIPA1L2, SIPA1L3, THBS1, TLN1, TLN2, VAV1	THBS1, PDGFRB, MRAS, NGFR, PDGFRA
Regulating pluripotency of stem cells	ACTN1, AKT3, CAPN2, COL1A1, COL1A2, COL4A1, COL4A2, COL6A1, COL6A2, COL6A3, COMP, EGF, FLNA, FLT4, FYN, IGF1R, ITGA11, ITGA2, ITGA5, ITGA6, ITGB3, ITGB5, JUN, KDR, LAMA2, LAMB2, LAMC1, LAMC3, MYL9, MYLK, PARVA, PARVB, PDGFC, PDGFD, PDGFRA, PDGFRB, PIK3R1, PIK3R3, PPP1R12B, PXN, RAPGEF1, RELN, SHC3, SHC4, SOS1, THBS1, THBS2, TLN1, TLN2, VCL, VWF, ZYX	PIK3R1, FZD4, INHBB, WNT4, IGF1R
