# Supplementary material for: Using an Unbiased Coexpression Network to Reveal Cross‐Talking Pathways of Phosphoinositide‐3‐Kinase Regulatory Subunit 1 in Skin Aging and Rejuvenation
Source: FASEB J. 2026 Jan 16;40(2):e71466. doi: 10.1096/fj.202402347RRRR (PMC12811739; doi:10.1096/fj.202402347RRRR)
Supplement: Supplementary file 7 — Data S1: fsb271466‐sup‐0007‐DataS1.pdf. [file FSB2-40-e71466-s003.pdf]

Statements

For our manuscript entitled "Using an unbiased coexpression network to reveal cross-talking pathways of phosphoinositide-3-kinase regulatory subunit 1 (PIK3R1) in skin aging and rejuvenation" (manuscript# 202402347R), which was submitted to FASEB Journal. The original author number and order is "Zhike Zhou[1, †], Sha Sha[1, †], Chundi He[2, 3], Ting Xiao[2, 3], Fenqin Chen[1, *], Le Qu[2, 3, *], Hong-Duo Chen[2, 3]". As request of the reviewers, we added some experiments. According to the authors' contribution, the revised author number and order is "Zhike Zhou[3, †], Sha Sha[3, †], Xiangnan Zhou[4, †], Chundi He[1, 2], Ting Xiao[1, 2], Yan Wu[4], Fenqin Chen[3, *], Le Qu[1, 2, *], Hong-Duo Chen[1, 2].

All the authors are aware of the new authors' contributions to the article, agree to the addition of the new authors, have been informed of the changes in author order, and agree with the new order arrangement.

Thank you so much for your consideration,

Yours sincerely,

Chundi He   Chen, Hong-duo

zhike zhou   Yan Wu.   Ting Xiao

Le Qu   Fenqin Chen

Sha Sha   Xiangnan Zhou
